# Phenotype-Driven Molecular Genetic Test Recommendation for Diagnosing Pediatric Rare Disorders

**DOI:** 10.21203/rs.3.rs-3593490/v1

**Published:** 2023-11-22

**Authors:** Fangyi Chen, Priyanka Ahimaz, Kai Wang, Wendy K. Chung, Casey Ta, Chunhua Weng, Cong Liu

**Affiliations:** 1 Department of Biomedical Informatics, Columbia University, New York, NY, USA; 2 Department of Pediatrics, Columbia University, New York, NY, USA; 3 Institute of Genomic Medicine, Columbia University, New York, NY, USA; 4 Raymond G. Perelman Center for Cellular and Molecular Therapeutics, Children’s Hospital of Philadelphia, Philadelphia, PA, USA; 5 Department of Pediatrics, Boston Children’s Hospital, Harvard Medical School, Boston, MA, USA

## Abstract

Rare disease patients often endure prolonged diagnostic odysseys and may still remain undiagnosed for years. Selecting the appropriate genetic tests is crucial to lead to timely diagnosis. Phenotypic features offer great potential for aiding genomic diagnosis in rare disease cases. We see great promise in effective integration of phenotypic information into genetic test selection workflow. In this study, we present a phenotype-driven molecular genetic test recommendation (Phen2Test) for pediatric rare disease diagnosis. Phen2Test was constructed using frequency matrix of phecodes and demographic data from the EHR before ordering genetic tests, with the objective to streamline the selection of molecular genetic tests (whole-exome / whole-genome sequencing, or gene panels) for clinicians with minimum genetic training expertise. We developed and evaluated binary classifiers based on 1,005 individuals referred to genetic counselors for potential genetic evaluation. In the evaluation using the gold standard cohort, the model achieved strong performance with an AUROC of 0.82 and an AUPRC of 0.92. Furthermore, we tested the model on another silver standard cohort (n=6,458), achieving an overall AUROC of 0.72 and an AUPRC of 0.671. Phen2Test was adjusted to align with current clinical guidelines, showing superior performance with more recent data, demonstrating its potential for use within a learning healthcare system as a genomic medicine intervention that adapts to guideline updates. This study showcases the practical utility of phenotypic features in recommending molecular genetic tests with performance comparable to clinical geneticists. Phen2Test could assist clinicians with limited genetic training and knowledge to order appropriate genetic tests.

## Introduction

Rare diseases each are uncommon conditions affecting fewer than 200,000 people in the United States^1–3^ but collectively affecting a large number worldwide^4^. The formal definitions of rare diseases differ slightly across countries as conditions that are more prevalent in one region may be rarer in other areas^5^. Individuals with rare diseases often endure a long diagnostic odyssey^4,6^, meeting with various disease specialists, undergoing numerous medical tests and procedures, and sometimes receiving misdiagnoses or inconclusive results along the way. Diagnostic delay can inevitably result in tremendous anxiety and financial burdens for patients and their family, missed treatment opportunities, compounding with disease progression^6,7^. Misdiagnosis can lead to inappropriate care that can be costly and associated with side effects. There are several well-established factors associated with delays in diagnoses. Most patients have limited access to experts with in-depth knowledge in recognizing and managing rare diseases^8,9^. Additionally, rare diseases marked by heterogenous symptoms further confuses which subspecialists to consult^6,7,10^. Consequently, patients receive multiple unnecessary referrals that often involve various specialists and diagnostic tests^11^.

A significant of portion of rare disease (50% - 75%) present initially in children^12^, with approximately 80% of rare diseases having a genetic basis^13^. Consequently, genetic testing is often regarded as the gold standard for yielding a definitive diagnosis. Currently, three main categories of genetic testing are adopted by most healthcare systems and available in genetic testing laboratories: i.e., cytogenetic, biochemical, and molecular testing^14^. Cytogenetic testing (e.g., microarray-based testing and karyotype) entails the examination of chromosomes for abnormalities, whereas biochemical testing focuses on the analysis of enzymes or metabolites^14^.

Molecular testing has emerged as a central approach for diagnosing genetic disease by sequencing DNA for mutations in one or more genes^15^. It offers high resolution and accuracy in identifying genetic abnormalities^16^. With molecular genetic testing, two options are commonly offered: whole-exome sequencing (WES)/whole-genome sequencing (WGS) or gene panels^15,17^. While WES/WGS can provide more comprehensive genome-level testing, these tests typically come with much higher costs and longer turnaround^18,19^. Gene panels can be a viable alternative to reduce expenses and enhance effectiveness for certain clinical indications. Gene panels are most effective when the disease-causing genes are known and included in the panels^18,19^. Either excessive or inadequate utilization of genomic tests (i.e. WES/WGS) can diminish the benefits for patients and impact the cost-effectiveness of testing^20–22^. Thus, an appropriate selection of molecular genetic tests is critical to obtain early, accurate genetic diagnoses, alleviating burden on patients, health care providers and society.

The current decision regarding the selection of WES/WGS and gene panels is mostly based on clinicians’ assessment and judgement^20,23^. A systematic approach or comprehensive guideline regarding the selection of genetic tests for rare disease diagnosis during clinical practice is underdeveloped^9,24^. A large disparity is often perceived in adopting and ordering genomic tests, strongly associated with physicians’ preferences and level of knowledge and understanding about available tests^20,24^. While utilizing comprehensive phenotype information for variant interpretation for WES/WGS analysis is well-established^25–28^, methods for selecting optimal diagnostic genetic testing based on patients’ phenotypic features is not well established^19^. Electronic Health Records (EHRs) are home to rich and nuanced clinical phenotypic data and promise to enable phenotype-driven analytical models^26,27^. Pipelines have been developed to extract phenotypic features from the EHR, thereby enriching the understanding of the relationship between genetic diseases and phenotypes^29^. Large consortiums such as eMERGE have leveraged the EHR phenotypes and their linked biobank genetic data to conduct genome-wide association studies^30^. Previous findings^31,32^ have suggested a significant difference in phenotypic features between control groups and individuals with Mendelian diseases, highlighting the potential of using phenotypes in facilitating the identification of patients with genetic diseases. Morley et al. demonstrated the utility of phenotypic features extracted from structured EHR billing codes in identifying individuals suitable for genetic testing^33^. Building upon their work, this study aims to develop a clinician-facing, phenotype-driven molecular genetic test recommendation (Phen2Test) for pediatric rare disease diagnosis. There is a great need for such a tool since most clinicians do not have knowledge of genetic tests. Our tool will aid such clinicians in selecting the appropriate molecular genetic test orders.

## Methods

**Figure 1.**
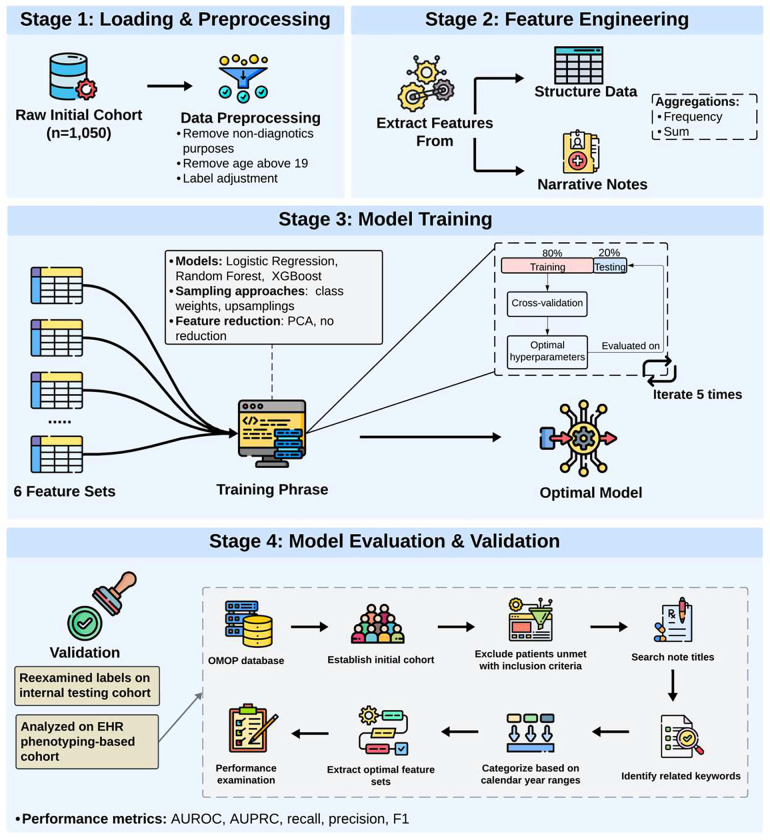
Study Overview. The study mainly consists of the four stages. *Stage 1: Loading and preprocessing*. This process involved feature engineering, model training and model evaluation and validation. During the data preprocessing stage, non-diagnostic purposes and individuals with age above 19 at the index date were excluded. *Stage 2: Feature engineering.* Features were extracted from both structure and narrative notes, with aggregation using two methods (frequency and sum). *Stage 3: Model training.* 6 different feature sets were passed into 3 classifiers, and models with the highest average AUPRC would be selected as the optimal model. *Stage 4: Model evaluation & validation*: two evaluations were performed on our optimal model (1) manual reexamination of the labels on a randomly selected cohort based on the initial testing cohort; (2) performance analysis on overall EHR cohort identified based on phenotyping method).

The study developed binary classification models by leveraging phenotypic features extracted from the electronic health records (EHR). The model’s output is either gene panel (negative class) or WES/WGS (positive class). An overview of the study design is depicted in Figure 1 and further elaborated in the subsequent sections.

### Data Description & Preprocessing

The initial dataset consisted of 1,005 rare disease patients who visited Columbia University Irving Medical Center (CUIMC) for potential genetic evaluation between 2012 and 2023. Given the scope of this study, individuals with genetic tests ordered for non-diagnostic purposes (e.g., gene panels ordered for pharmacogenomics analysis), or recurrence risk prediction in a family with an established mutation were also excluded. We further narrowed the inclusion criteria to only focus on the pediatric setting by excluding individuals whose age at the first appointment date was 19 years or older. The test order status of the patients’ genetic tests was recorded manually into a research database (REDCap) and categorized into three groups: patients who received (1) whole-exome sequencing/whole-genome sequencing (WES/WGS) only, (2) gene panel-based variant detection (gene panels) only, or (3) gene panels first, followed by WES/WGS due to negative panel results. In this study, we considered the third group as WES/WGS instead since they ultimately received genomic sequencing. The clinical decisions regarding genetic test ordering were made through a comprehensive assessment that considered various factors. The microarray-based tests was not considered in this cohort because WES/WGS was perceived to offer significantly greater clinical and diagnostics utility in testing children with suspected genetic diseases^34–36^ compared to chromosomal microarray (CMA)^37^. The study was approved by Columbia University Intuitional Review Board.

### Label Adjustment

Genetic testing recommendations and guidelines are dynamic and are constantly evolving. Given that we collected patient data from 2012–2023, it is important to note that decisions documented at the time of patient visits may not reflect the optimal testing choice today given the latest guidelines. We therefore manually adjusted the labels for optimal test orders based on the phenotype summaries and the latest literature for best genetic tests for the corresponding phenotypes. These phenotype summaries were provided in the initial curated genetic dataset, where clinicians documented key phenotype indicators by reviewing patients’ previous records. We then harmonized these findings in alignment with the updated guidelines. According to current recommendation by American College of Medical Genetics and Genomics (ACMG)^38^, WES/WGS can be considered as a first-tier test for patients with congenital anomalies, developmental delay, intellectual disability, neurological developmental disability (e.g., autism spectrum disorder, attention-deficit/hyperactivity disorder)^39,40^, or seizures^41,42^. Genetic test labels were updated to WES/WGS if any of the above conditions were entailed in the summary. It is important to note that the phenotype summary was manually recorded in the research database, separate from the original EHR data (used for model training), and was not directly utilized as features for model training. Labels before and after adjustment were utilized to train the models separately, and performances were documented and compared.

### Feature Engineering

In this study, we extracted phenotypic features from both structured and unstructured data. Since the recommendation system is intended for use during the test ordering process, we exclusively focused on individual’s EHR data before the index date, established as their respective test date. In cases when the test date was unavailable, we used the genetic appointment visit date as the index date, assuming negligible gaps between the two, thus allowing for their interchangeable use. A list of features is outlined in Table 1. The process of feature extraction is detailed below.

#### Feature Extraction: Structure Data

We extracted the patients’ medical conditions from the Observational Medical Outcomes Partnership (OMOP) database, which uses a standardized database schema and set of terminology mappings for integrating diverse healthcare data sources from different EHR systems for research and analysis. The obtained conditions were first converted from standard OMOP concept to ICD10 CM and mapped to the corresponding phecodes (version 1.2). Phecodes are primarily designed for phenome-wide association studies (PheWAS)^43,44^, and have been used to train classification models for various clinical genetic tasks^33^. Afterwards, we implemented two data aggregation approaches of the identified phecodes per individual, such as (1) the frequency of each identified phecode (*Freq_phecodes*), and (2) sum of unique phecodes (*Sum_phecodes)*. To avoid the redundancy introduced during the concept mapping, we opted to count the phecodes only once if two or more identical phecodes were observed on the same date for a given individual. Besides phecodes, Human Phenotype Ontology (HPO)^45^ is another standard reference for describing phenotypic abnormalities and has been adopted by various rare disease organizations, clinical labs, biomedical resources, and clinical software utilities^46^. We constructed an additional feature set by mapping OMOP condition concepts to HPO terms followed by the categorization of HPO terms according to their underlying *Phenotypic abnormality* (e.g., abnormality of the musculoskeletal system). The counts of each HPO-based organ systems of phenotypic abnormality (n=23) were subsequently utilized as input features (*Freq_HPO)*. Demographics characteristics (sex, race, and age at the index date) as well as different types of patient care (i.e., in-patient or out-patient) at the index date were also extracted and incorporated into the modeling process.

#### Feature Extraction: Unstructured Data

We applied regular expressions to identify additional phenotypic features from clinical narratives that matched with the phecodes system. Additionally, we employed a trained language model developed by Aken et al. for negation and scope detection (F1 score of 0.972 in detecting negated terms on i2b2 discharge summaries^47^), only including phenotypes observed in the individuals. Following a similar data aggregation approach described above, we calculated the frequency of each identified phecode based on extracted results (*Freq_phecodes_notes)* and the sum of uniquely identified phecodes (*Sum_phecodes_notes*). In addition, we derived the cumulative count of clinical notes documented prior to the index date (*Num_notes)*, where these combined factors (age, frequency of healthcare utilization, presented phecodes) were leveraged as a proxy to reflect the severity of a patient’s condition.

### Model Training & Evaluation

We generated multiple feature sets, incorporating combinations of the previously mentioned attributes, and trained them using three different classifiers: Logistic Regression, Random Forest, and XGBoost. A nested cross-validation was performed for hyperparameter optimization and model evaluation, involving a three-inner fold cross validation on 80% of the entire dataset and the remaining 20% was reserved for testing. This partitioning was performed while maintaining the consistent distribution of prediction labels (types of genetic test). The models with the optimized hyperparameters achieving the highest F1-score on validation set were then selected and evaluated on the testing set. We also evaluated different strategies in mitigating class imbalance issue, including class weight adjustment, upsampling using SMOTE^48^ or random duplication of data points from the minority class. Furthermore, we performed principal components analysis (PCA) and evaluated the impact of feature reduction on classification performance. The process went through five iterations for different sets of features, where performance was recorded at each iteration and averaged in the end. The evaluation metrics (precision, recall and F1-measure) were calculated using a threshold of 0.5, along with the area under the ROC curve (AUROC) and precision-recall curve (AUPRC). The combination achieving the highest averaged AUPRC was considered as the optimal model and was used for further analysis.

### Manual Inspection & Validation

Genetic test decisions often consider economic and financial factors, patient preferences, insurance, and current technology limitations^20^. Factors like genetic expertise, education, and institutional policies also impact test choice^9^. The main objective of this study was to construct a recommendation system based solely on EHR-documented phenotypes/conditions to provide decision support purely from a clinical perspective. Therefore, to mimic a test environment where only clinical factors are considered in the choice of genetic test, a genetic counselor independently chart-reviewed a group of randomly selected individuals (n=30) from the test set.

The objective was to verify whether the initial test recommendations were solely based upon the reported phenotypes/conditions and were consistent with current practice guidelines. Any observed misalignments were captured and corrected. We performed 200 iterations of bootstrapping on the 30 reevaluated labels, sampling with replacement, and for each iteration, we computed performance metrics AUROC and AUPRC according to the reevaluated labels. Performance was documented for each iteration and averaged at the end of the run. The 95% confidence interval was constructed using the calculated mean and standard error, with a critical value of 1.96, presenting a more reliable representation of the model’s performance.

#### Analysis on a Silver Standard Cohort

We further analyzed our model on a larger cohort in the OMOP database using a phenotype-derived approach^49^. We first identified a “genetic” cohort from the structured database as individuals who had visited the genetic clinics, undergone genetic tests, or had genetic related measurement analysis (a complete list of OMOP concepts is provided in **Supplemental Table 1**) between 2012 and 2023. Next, we excluded patients whose age at the date of genetic appointment/measurement was 19 years or older. Afterwards, we implemented a two-step process to identify the types of genetics tests recommended for each patient: 1) identified clinical notes that contained the keywords such as “genetic”, “letter”, “visit”, “progress note” in their titles, 2) further identified notes that contained keyword “panel”, “exome”, “genomic”, “WES”, or “WGS”. Some consideration was taken to improve the identification of the “panel” group. This involved excluding cases related to biochemical testing, viral panel testing, routine blood panel, or for screening purposes. A complete list of keywords/regular expressions is provided in **Supplemental Table 2**. The label extraction algorithm was evaluated on our initial testing cohort where annotations were available, achieving an accuracy of 89% in identifying labels (after adjustment). While recognizing the possibility of discrepancies between actual ordering and labels extracted from notes, this phenotype-derived cohort served as a reliable substitution when the manual label annotation was not feasible for the large scale of data. Finally, visit dates associated with the notes when the keywords were identified were used as the index dates. For individuals who had both WES/WGS and gene panel tests documented, we considered the former as the label, with the index date being the earliest date when the testing recommendation was made. To reflect the evolving clinical practice trend, the eligible individuals (n=6,458) were divided into four calendar year ranges according to their index dates: 2012–2014, and 2015–2018, 2019–2021 and 2021–2023. The same feature extraction procedure described above that yielded the best performance in the held-out testing dataset was implemented to extract features. The trained model was then employed to examine the influence of temporal variability on model performance.

### Results Interpretation

Regarding the feature importance, we quantified the significance of phenotypes in the main prediction model using Gini impurity criterion^50^, where each feature importance was calculated as the mean decrease in Gini impurity specifically referring to normalized total impurity reduction values for nodes where splitting was based on that feature^51^. The correlation between the types of phenotypic abnormalities and labels (WES, panel) was quantified using ordinary least squares (OLS) regression technique, from which we derived the corresponding odd ratios (ORs) and p-values. Moreover, the phecodes were clustered into 17 groups based on PheWAS categorization (version 1.2) to gain a system-level perspective on how phenotype abnormalities in various organ systems influenced the test recommendations. This phecode-based finding was further juxtaposed with an alternative HPO ontology to identify any discrepancies related to the choice of the standard vocabulary for feature representation.

## Results

### Data Characteristics

The demographic characteristics of the initial manually curated cohort and the large phenotyping-based cohort extracted from the OMOP database are provided in Table 2. In the initial cohort (n=1,005), 37.1% of the patients were White. Prior to label adjustment, there were 570 individuals recommended for WES/WGS, and 435 for gene panels. After the label adjustment, 139 individuals who were originally recommended for panels were shifted to WES/WGS (296 gene panels, 709 WES/WGS). The mean ages of the patients recommended for WES/WGS were 4.92 (std: 4.87) and 4.88 (std: 5.00) years before and after label adjustment, respectively, while mean ages of the gene panel group were older (before correction: 5.50 years, after correction: 5.88 years). The age difference was also observed in the larger cohort (n=6,458), with a mean age of around 5.56 years in WES/WGS group and 10.05 years in the panel group. It is important to note that in the larger cohort, based on the extraction results, the number of panel cases (n=3,427) exceeded the number of WES/WGS cases (n=3,031).

### Performance Evaluation on Initial Cohort

**Figure 2.**
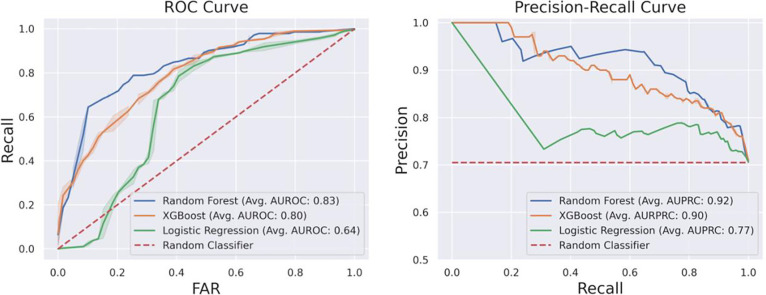
ROC Curve (left) and Precision-recall Curve (right) Derived from the Initial Curated Testing Cohort. Receiver operating characteristic (ROC) curves and Precision-Recall curves were used to illustrate the performances of the optimal feature sets across three classifiers on the held-out testing dataset from the initial cohort. The shaded area represents the confidence interval calculated from iterative results.

After experimenting with different feature sets and sampling strategies, the model achieving the highest average AUPRC (0.918, std: 0.023) was Random Forest built on features including phecodes aggregated by frequency derived from structured data, demographics characteristics and the number of notes. It applied class weight adjustments to address class imbalance and did not perform feature reduction. The ROC curve (average AUROC 0.822) and precision-recall curve are depicted in Figure 2. As shown in Table 3, there was ~ 10% improvement in all performance metrics after the label adjustment. When trained and evaluated using raw annotated labels, the average AUPRC was 0.811 (std: 0.022), with an average AUROC of 0.781 (std: 0.024). Phecode-based feature sets yielded better performance compared with features derived from HPO ontology, as shown in Table 4. Additionally, we did not perceive a meaningful gain in the performance while leveraging phenotypes extracted from both structured data and narrative notes. The performance of all trained models across various features, strategies and classifiers is in **Supplemental Table 3**.

### Manual Inspection of the Initial Cohort

Clinicians reviewed randomly sampled cases (n=30) from the test cohort and assigned recommended genetic test labels based on phenotypes only. Out of the 30 instances, 3 testing labels (10%) were different from the newly annotated ones. Among those, two (2/3) of the altered cases were switched from gene panels to WES, with primary indicator diseases being retinal diseases and congenital myasthenic syndromes (CMS), and sensorineural hearing loss (SNHL). One case was altered from WES to panel, with the primary disease indicated as obesity. The reannotated labels were regarded as the most ideal genetic tests based solely on phenotypes, which would be evaluated alongside our model predictions. The 95% confidence interval for performance metrices was computed through performing 200 iterations of bootstrapping. As compared with the previous results (label adjusted for guidelines) shown in Table 3, the average AUROC of reannotated labels was estimated to be 0.887 (95% CI: 0.877 – 0.896), and the average AUPRC was computed as 0.972 (95% CI: 0.970 – 0.975). The model yielded better prediction outcomes when evaluated on reannotated labels adjusted for phenotypes, in contrast to the previous recommended results (Table 3) where such outcome labels were influenced by various external factors.

### Analysis of EHR Phenotyping-based Cohort

We performed additional validation of the trained model on the larger, silver standard cohort (n=6,458) derived from the OMOP database, where about 46.9% of patients were recommended to receive WES/WGS. Across all calendar years, we perceived an overall AUROC of 0.718 and AUPRC of 0.671. Compared with the curated genetic testing cohort, both performance metrics exhibited a reduction of more than 10% on the EHR phenotyping-based cohort. As our model was trained based on the labels assigned according to the current guideline, we observed a discernible trend of performance improvement as the time approached more recent dates, with the AUROC increasing from 0.684 in 2012 to 0.76 in 2020 (Table 5). The drop in performance post-2021 was likely caused by shifts in healthcare behavior due to the pandemic which significantly disrupted traditional clinical practices^52^.

### Analysis of feature importance

**Figure 3.**
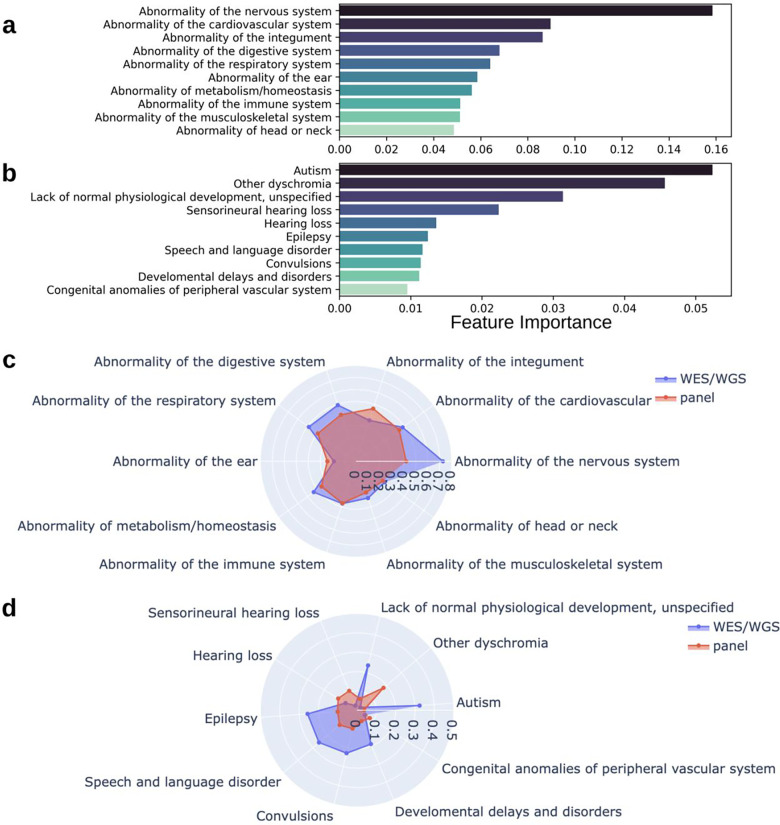
Most Predictive Phenotypes and Phenotype Categories. The top 10 feature importance scores calculated by Gini impurity for both (**a**) HPO phenotypic abnormalities (n=23), and (**b**) Phecodes (n=1,225). Two radar charts illustrate the proportion of (**c**) the top 10 HPO phenotypic abnormalities, and (**d**) Phecodes within two different groups: WES/WGS, panel.

The feature importance of phenotypic abnormalities under both HPO ontologies and phecodes were computed using Gini impurity criteria. The top 15 important features were visualized in Figure 3. We perceived identical feature importance outcomes between HPO ontology and Phecode phenotypes. Systems of phenotypic abnormalities significantly (p-value <0.05) associated with genetic test decision-making included nervous system, growth abnormality, limb abnormality, genitourinary system abnormality, all of which were positively correlated with WES/WGS ordering. Abnormality of the digestive system showed a negative correlation with WES/WGS test. Furthermore, we clustered phecodes based on PheWAS ontology to investigate the important phecodes categories where individual important phecodes were overrepresented. We conducted a Chi-square independent test to identify highly significant categories (adjusted p-value < 0.01), including neurological, mental disorders, metabolic, sense organs and dermatologic (See **Supplemental Table 4** and **Supplemental Table 5** for the complete list).

## Discussion

Individuals with rare diseases often encounter prolonged diagnostic delays, highlighting a need to optimize the genetic test ordering process to ensure timely diagnosis and effective disease management. A previous study has showed that shared phenotypes among patient groups can be leveraged to identify individuals likely to benefit from CMA testing with relatively high accuracy^33^. Although not shown in our results, by adopting a similar approach and applying it to our cohort, we achieved nearly identical performance with an AUROC and AUPRC of 0.97 and 0.97, respectively. With molecular genetic testing becoming the gold standard for genetic diagnosis, our study further advances the genetic test ordering process by providing additional recommendations to ordering physicians for selection of appropriate molecular genetic/genomic tests. Thus, our findings enhance a future workflow towards practically using the phenotypic features to more systematically diagnose rare genetic disorders with decision support to providers. Universal decision support could help decrease disparities to obtaining a genetic diagnosis.

The model achieved its highest performance when utilizing features derived from structured billing codes (converted to Phecodes) along with basic demographic information. While various studies^26,53,54^ have explored the extraction of clinical phenotypes from clinical narratives for genetic data analysis, our study did not exhibit any noticeable performance improvement when using phenotypes extracted from clinical notes. Additionally, we also explored the use of phenotypes based on another popular ontology, HPO, by extracting HPO terms from the notes, as described in a previous study^29^, and we found no statistical improvement when using either high-level HPO phenotypic abnormality or fine-grained HPO terms. While this lack of improvement may be due to the simplicity of our phenotype extraction algorithm (e.g., largely based on keyword search), another possible explanation is that the information necessary for predicting genetic test ordering is already effectively summarized in high-level structured data. For example, we showed phenotypes involved with nervous system, growth abnormality, limb abnormality, genitourinary system abnormality can contribute to the decision to order WGS/W, and these systemic abnormalities can be readily inferred from the ICD codes. Furthermore, our recommendation aligned with established clinical guidelines, such as recommending WES/WGS for conditions like congenital anomalies, developmental delay, intellectual disability, neurological developmental disability, all of which are likely to be clearly summarized within the structure data.

Extracting comprehensive lists of phenotypes from narrative notes remains a challenging task, often demanding substantial manual annotation by domain experts to construct suitable extraction algorithms^55^. Recent studies^56^ have explored leveraging existing ontologies along with pre-trained transformer models to extract phenotypes from text. Given the recent advancement of large language models (LLM) and its potential in diverse NLP tasks with few-shots of training^57^, researchers have^58^ demonstrated the utilization of a GPT-based model to enhance the detection of phenotype terms from clinical notes. However, we noticed many phenotypes cannot be directly represented by HPO vocabulary, and given the capability of LLM in comprehending narratives, it prompts a new question - do we still need to extract those intermediate-level concepts to help with downstream predictions? While further research is required to explore the potential use of a fine-tuned LLM for directly conducting these prediction tasks, it is important to note the fine-tuning or training process may involve sensitive data, and there is a risk that the model could inadvertently retain information from the training context and disclose it in a subsequent prediction task^59^.

WES/WGS were initially introduced into the clinical settings in 2012^60^, a time when both genomic testing and supporting evidence were limited^61^. As more evidence emerged and clinical guidelines evolved, an increasing number of physicians began to incorporate WES/WGS into their diagnostic assessments. Our model, specifically trained and adjusted for the latest clinical guidelines, effectively reflected this temporal trend – exhibiting improved performance in more recent cohorts. This underscores its potential as a component of a learning health system^62^, capable of direct training using the EHR data, facilitating the dynamic updating of clinical guidelines, and consequently offering evidence-based decision support to healthcare providers. Moreover, while WES/WGS has been adopted for newborn screening^63^, the limited phenotypes present at birth has posed challenges for the WES/WGS data analysis at prenatal or neonatal stages^64^. It has been showcased by many publications^65,66^ the benefits of re-analyzing sequencing data in the light of evolving knowledge and understanding of the molecular aspects of medical conditions. The determination of appropriate timing for re-analyzing genomic data based on individuals’ conditions is unclear^67^. Phen2Test is primarily designed for the selection of genomic/genetic tests based on the latest EHR data, which can also enable the identification of the “index date” – determining when the WES/WGS data (re)analysis is needed for children by analyzing their most up-to-date phenotypes documented in the EHR. In other words, our model is applicable to the selection of virtual panel and WES/WGS driven by phenotypic conditions.

Several concerns need to be addressed before deploying this system into a routine clinical workflow. First, we observed a higher ratio of WES/WGS in both our initial curated cohort and larger phenotyping-based cohort. This may be attributed to the fact that our study was conducted within a high-resource healthcare institution (i.e. an academic medical center) where physicians are more likely to be well-informed about and inclined to order advanced, cutting-edge tests. Hence, it is essential to acknowledge that the system’s generalizability may be limited when applied to lower-resource healthcare systems, as the training data might not accurately represent the true distribution within the intended cohort. Unfortunately, this challenge is a pervasive limitation encountered in many machine learning or AI-based approaches, where models are often trained within high-resource academic centers but are increasingly sought after in low-resource clinical settings, such as rural pediatric clinics^68^. While the relatively simple billing-codes based feature engineering pipeline used in our study might help enhance adaptability and retraining capabilities across various systems, how to address this resource imbalance remains a significant and pressing concern.

Furthermore, many other factors such as financial costs and resource availability can also be relevant in the real-world decision-making process^9,20^. While Phen2Test does not account for those external factors, the question arises hypothetically whether clinicians should always choose WES/WGS if these constraints were eliminated. Despite the fact that WES/WGS has higher yield in detecting pathogenic variants, they also are associated with incidental findings that many non-geneticists do not feel comfortable managing^69^. Additionally, a previous study^70^ has shown that clinical WES/WGS may not fully capture all exons, detect mosaicism, or detect small intragenic deletions/duplications in clinically implicated genes. This suggests that a well-covered disease focused gene panel could be a more suitable choice when the phenotypes clearly indicate an underlying genetic disease. On the other hand, payer barriers have limited the clinical use of WES for patients with suspected genetic diseases^71,72^, with reimbursement sometimes denied^73^. The outcomes generated by Phen2Test can provide more objective decisions and gather more data to assess the medical impact of performing specific genetic tests.

## Conclusion

This retrospective study develops and validates an effective phenotype-driven approach to identifying suitable molecular genetic tests for diagnosing pediatric rare disorders. The approach can potentially assist clinicians with minimum genetic tests knowledge in ordering relevant tests. More field studies are warranted to test the effectiveness of this approach prospectively in clinical settings and the generalizability to different EHR systems.

## Figures and Tables

**Table 1.** Features used to train the classification models.

Categories	Feature Names & Description	Input Feature Dimension

**Clinical Features (Structured Data)**	- *Freq_phecodes*: frequency of each phecodes/phenotype	1,225
	- *Sum_phecodes*: total number of unique phecodes/phenotypes	1
	- *Freq HPO*: frequency of each HPO-based organ systems of phenotypic abnormality	23

**Clinical Features (Unstructured Data)**	- *Freq_phecodes notes*: frequency of each phecodes/phenotypes derived from clinical narratives	418
	- *Sum_phecodes notes*: total number of unique phecodes/phenotypes derived from clinical narratives	1
	- *Num notes*: cumulative sum of notes	1

**Demographics Characteristics**	- *Age*	1
	- Sex assigned at birth time	1
	- *Race self-reported by patients*	1

**Table 2.** Demographics characteristics of initial manual curated and larger phenotyping-based cohorts. WES/WGS: whole-exome sequencing/whole-genome sequencing. Panel: gene panel.

Demographics Characteristics	Clinicians–Curated Genetic Cohort (n=1,005)		EHR Phenotyping-based Cohort (n = 6,458)	
	WES/WGS^a^	Panel^a^	WES/WGS^b^	Panel^b^

**Race**				
White	271 (227)	102 (146)	1447	1642
Black or African American	79 (58)	34 (55)	344	363
Asian	30 (25)	9 (14)	116	78
Other (e.g., American Indians or Alaska nation)	2 (2)	4 (4)	504	42
Not described	176 (139)	79 (116)	42	592
Decline or not specified	151 (119)	68 (100)	578	710

**Sex**				
Male	452 (362)	169 (259)	1687	1502
Female	257 (208)	127 (176)	1342	1924
Other	-	-	2	1

**Age at the index date**				
Mean age ± Std	4.88 ± 4.87 (4.92 ± 5.00)	5.88 ± 5.91 (5.50 ± 5.46)	5.56 ± 5.53	10.05 ± 6.09
Median age	3.48 (3.46)	3.65 (3.61)	5.27	10.87
0 – 5	443 (359)	165 (249)	1470	914
5 – 10	140 (103)	52 (89)	722	675
10 – 15	93 (79)	47 (61)	505	837
>= 15	33 (29)	32 (36)	334	1001

**Total WES/WGS cases**	709 (570)	296 (435)	3,031	3,427

a Genetic testing labels annotated by clinicians, followed by the label counts after adjustment (statistics before label adjustment).

b Genetic testing labels extracted from clinical narrative notes based on phenotyping approach.

**Table 3.** Average performances (std.) of the optimal predictive model based on the held-out testing sets in the initial cohort. Raw annotated labels referring to initial labels manually entered by clinicians before adjustment. Those labels were later adjusted to meet current clinical guidelines for whole exome sequencing and whole genomic sequencing test ordering.

Model	Recall	Precision	F1	Accuracy	AUROC	AUPRC
Random Forest (raw annotated labels)	0.842 (0.022)	0.728 (0.016)	0.780 (0.011)	0.731 (0.015)	0.781 (0.024)	0.811 (0.022)
Random Forest (After label adjustment)	0.941 (0.024)	0.801 (0.021)	0.865 (0.019)	0.793 (0.029)	0.822 (0.045)	0.918 (0.023)

**Table 4.** Average performances across different feature sets based on the held-out testing sets in the initial cohort after label adjustment.

Feature Sets	Feature Dimension	Average AUROC	Average AUPRC
Phecodes [structured] + Demographics	1228	0.831 ± 0.040	0.917 ± 0.022
HPO + Demographics	26	0.737 ± 0.051	0.852 ± 0.042
Phecodes [unstructured] + Demographics + note counts	422	0.803 ± 0.049	0.90 ± 0.025
**Phecodes [structure] + Demographics + note counts**	1229	0.822 ± 0.045	**0.918 ± 0.023**
HPO + Demographics + note counts	27	0.736 ± 0.054	0.864 ± 0.035
Phecodes [structure & unstructured] + Demographics + note counts	1297	**0.838 ± 0.052**	0.917 ± 0.028

**Table 5.** Performance across different time periods based on the large phenotyping-based cohort.

Calendar Year Ranges	AUROC	AUPRC

2012 – 2014 (n=1,843)	0.684	0.585
2015 – 2017 (n=1,627)	0.695	0.655
2018 – 2020 (n = 1,775)	0.757	0.744
2021 and beyond (n= 1,213)	0.741	0.700

All years	0.718	0.671

## Data Availability

The code and the final trained model utilized in this study are available on GitHub at https://github.com/stormliucong/RARE-GOrder. The clinical data used in this study contains Protected Health Information (PHI) and, as such, cannot be made readily available for distribution. Requests for access to the data will undergo review by the institutional IRB (Institutional Review Board) for consideration.
